# Evidence of clinical and brain recovery in post-COVID-19 condition: a three-year follow-up study

**DOI:** 10.1093/braincomms/fcaf366

**Published:** 2025-09-23

**Authors:** Ravi Dadsena, Sophie Wetz, Anna Hofmann, Ana Sofia Costa, Sandro Romanzetti, Stella Andrea Lischewski, Christina Krockauer, Carolin Balloff, Ferdinand Binkofski, Jörg B Schulz, Kathrin Reetz, Julia Walders

**Affiliations:** Department of Neurology, RWTH Aachen University, 52074 Aachen, Germany; JARA Brain Institute Molecular Neuroscience and Neuroimaging (INM-11), Research Centre Jülich and RWTH Aachen University, 52056 Aachen, Germany; Department of Neurology, RWTH Aachen University, 52074 Aachen, Germany; German Center for Neurodegenerative Diseases (DZNE), 72076 Tübingen, Germany; Department of Cellular Neurology, Hertie Institute for Clinical Brain Research, University Hospital Tübingen, 72076 Tübingen, Germany; Department of Neurology, RWTH Aachen University, 52074 Aachen, Germany; JARA Brain Institute Molecular Neuroscience and Neuroimaging (INM-11), Research Centre Jülich and RWTH Aachen University, 52056 Aachen, Germany; Department of Neurology, RWTH Aachen University, 52074 Aachen, Germany; JARA Brain Institute Molecular Neuroscience and Neuroimaging (INM-11), Research Centre Jülich and RWTH Aachen University, 52056 Aachen, Germany; Department of Neurology, RWTH Aachen University, 52074 Aachen, Germany; JARA Brain Institute Molecular Neuroscience and Neuroimaging (INM-11), Research Centre Jülich and RWTH Aachen University, 52056 Aachen, Germany; Department of Neurology, RWTH Aachen University, 52074 Aachen, Germany; Department of Neurology, Medical Faculty and University Hospital Düsseldorf, Heinrich Heine University, 40225 Düsseldorf, Germany; Department of Neurology, Kliniken Maria Hilf GmbH, 41063 Mönchengladbach, Germany; JARA Brain Institute Molecular Neuroscience and Neuroimaging (INM-11), Research Centre Jülich and RWTH Aachen University, 52056 Aachen, Germany; Institute for Neuroscience and Medicine (INM-4), Research Center Jülich GmbH, 52425 Jülich, Germany; Department of Neurology, RWTH Aachen University, 52074 Aachen, Germany; JARA Brain Institute Molecular Neuroscience and Neuroimaging (INM-11), Research Centre Jülich and RWTH Aachen University, 52056 Aachen, Germany; Department of Neurology, RWTH Aachen University, 52074 Aachen, Germany; JARA Brain Institute Molecular Neuroscience and Neuroimaging (INM-11), Research Centre Jülich and RWTH Aachen University, 52056 Aachen, Germany; Department of Neurology, RWTH Aachen University, 52074 Aachen, Germany; JARA Brain Institute Molecular Neuroscience and Neuroimaging (INM-11), Research Centre Jülich and RWTH Aachen University, 52056 Aachen, Germany

**Keywords:** long COVID, fatigue, CNS, MRI, ALFF

## Abstract

Fatigue and cognitive dysfunction linked to persistent brain changes have been reported for up to two years after COVID-19. In this study, we followed the clinical, neuroimaging and fluid biomarker trajectories over three years post SARS-CoV-2 infection to evaluate potential signs and underlying factors of brain recovery. We conducted a monocentric, longitudinal study using resting-state functional and structural T1-weighted magnetic resonance imaging data from 51 patients with post-COVID-19 condition (mean age 50 years, 33 female) collected at a mean time of 6, 23 and 38 months after COVID-19 infection. The trajectory of brain changes was compared to 23 age- and sex-matched healthy controls (mean age 37 years, 13 female) with similar time intervals between brain scans and analysed in relation to clinical, neuropsychological and fluid biomarkers including interleukins and neurodestruction markers at all timepoints. In addition, hand grip strength to evaluate muscular fatigue was assessed at the final follow-up visit. Self-reported fatigue improved over time but was still moderate on average three years after COVID-19 infection, while measures of hand grip strength and cognitive performance were largely unaffected. We found a significant increase of both lateral ventricles (∼8%) and the third (∼6%) ventricle accompanied by a structural volume reduction in adjacent areas including the thalamus, pallidum, caudate nucleus and putamen. An increased neuronal activation pattern was widespread and pronounced in these areas. The brainstem no longer exhibited volume loss as reported in our pervious study, but enhanced functional connectivity. Laboratory markers including interleukins and neuronal injury markers remained within the normal reference ranges across all study timepoints. Our study revealed an overall slow but evident clinical improvement, including improved fatigue, regular muscular strength and recovery as well as normal cognitive function without signs of systemic inflammation three years after COVID-19. Clinical improvement is reflected by a pattern of brain recovery along periventricular regions. This pattern is characterized by structural stabilization and increased connectivity starting in the brainstem as well as efficient neuronal recruitment and increased activation in the basal ganglia, with no evidence of neuronal injury. These results highlight the positive long-term recovery trajectory in post-COVID patients.

## Teaser

Dadsena *et al*. report on brain changes in patients with post-COVID-19 condition three years after SARS-CoV-2 infection using multimodal MRI brain imaging, clinical, neuropsychological and fluid markers. They found that clinical improvement and brain recovery are slow but evident three years following acute COVID-19 without signs of ongoing systemic inflammation and neurodegeneration.

## Graphical Abstract

**Graphical Abstract:**
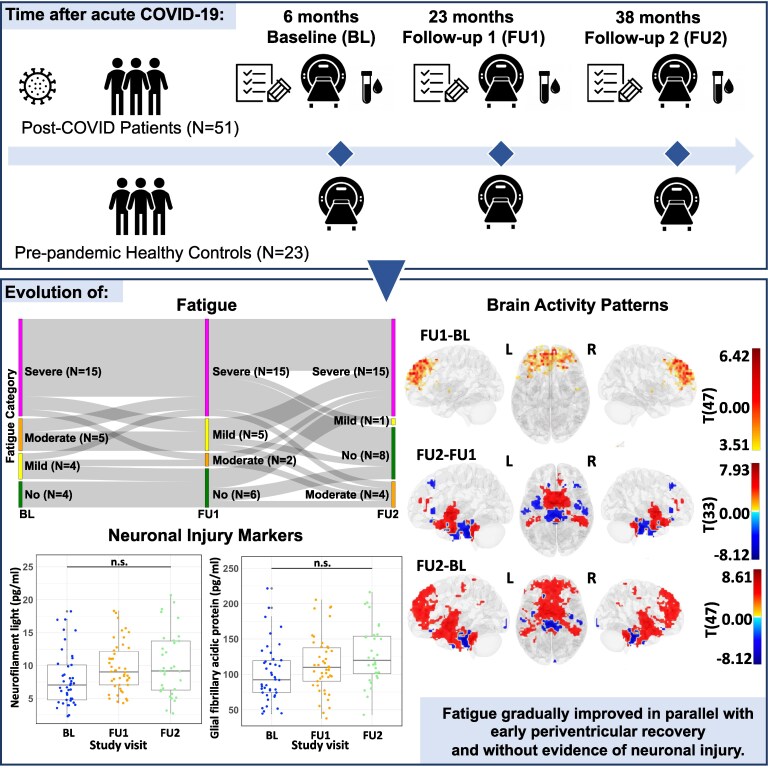

## Introduction

Following COVID-19 infection, 10–15% of individuals experience a post-infectious condition characterized by multisystemic symptoms, predominantly neurological in nature including cognitive complaints and fatigue.^1^

The majority of patients after COVID-19 only improve slowly, and 4% show persistent complaints two years after symptom onset.^2^ Limited to a temporal criterium of ongoing or new symptoms 12 weeks after SARS-CoV-2 infection, the current definition of post-COVID-19 condition (PCC) falls short to reflect the complexity of the disease and to distinguish between patients whose symptoms arise from neuronal and non-neuronal factors.

Multimodal neuroimaging studies on PCC patients have revealed microstructural, connectivity and perfusion abnormalities in PCC, including gray matter reduction, white matter tract injury, disrupted brain networks, choroid plexus enlargement, impairment of the blood-brain barrier and altered cerebral blood flow.^3-5^ In a previous, cross-sectional analysis after 6 and 24 months post-infection, we identified widespread brain changes involving the brainstem, the pre- and postcentral gyrus and the limbic olfactory network in PCC patients compared to healthy controls.^6^ Other studies also reported alterations in the brainstem, the cerebellum, the basal ganglia, the thalamus, the hippocampus and orbitofrontal areas to be associated with cognitive dysfunction and fatigue.^5,7-10^ However, longitudinal neuroimaging data are currently limited to 24 months post-infection and most studies lack assessment of inflammatory and neuronal injury markers, warranted to establish a conclusive disease concept for the brain changes observed. This may be of particular interest in PCC, as ongoing immune dysregulation is considered to be part of PCC pathogenesis^11^ and inflammatory markers including a specific cytokine triad have been proposed as distinguishing factors of PCC patients.^12,13^ However, these findings could not be replicated in another PCC cohort.^14^

Regarding neurodegeneration in PCC patients, studies on neurofilament light (NfL) and glial fibrillary acidic protein (GFAP) as biomarkers for neuronal damage and astrocytic activation yielded varied conclusions up to date. A longitudinal study found that 6 months post-infection, NfL and GFAP returned to normal levels despite ongoing fatigue and cognitive problems in many patients.^15^ Similarly, other studies in PCC patients found that persistent cognitive impairment in most subjects was not associated with neuronal and astrocytic damage.^16,17^ In contrast, another study reported hippocampal changes linked to cognitive dysfunction and elevated NfL and GFAP levels in PCC patients.^18^ Similarly, a recent study involving 63 PCC patients found that those exhibiting cognitive dysfunction and fatigue had elevated NfL levels compared to patients without these symptoms, highlighting a potential neurobiological basis for these manifestations.^19^ Overall, uncertainties remain regarding the diagnostic value of NfL and GFAP in relation to CNS changes and symptoms in PCC.

To shed light on the evolution and associations between clinical, biochemical and imaging trajectories in PCC patients, we conducted comprehensive neuropsychological assessments, multimodal imaging analyses on volumetric changes, neuronal activation and functional connectivity and extensive blood marker analyses in 51 PCC patients over a period of three years post-infection.

Based on current knowledge, showing overall clinical improvement in PCC and recurringly observed brain alterations, we hypothesize that clinical improvement continues and is linked to the neuronal restoration of previously reported regions.

## Materials and methods

### Study participants

In this monocentric, longitudinal, observational study initiated in August 2020, patients were recruited from the Department of Neurology at the Uniklinik RWTH in Aachen (UKA), Germany. Data from healthy controls without neurological or psychiatric comorbidities were used from research projects prior to the onset of the COVID-19 pandemic with identical imaging protocols and similar time intervals between scanning as included patients, that is, 6 months for baseline, 23 months for follow-up 1 and 38 months for follow-up 2. Eligibility criteria for the study included being 18 years or older with persistent neurological symptoms after confirmed SARS-CoV-2 infection. MRI exclusion criteria included contraindications such as metallic implants or claustrophobia. The study included 51 patients with PCC and 23 age- and sex-matched healthy controls. Although the PCC group had a mean age that was 13 years older than the control group, both groups were matched within the age range of 25 to 55 years to minimize age-related differences in brain structure and function.^20^

### Standard protocol approvals, registrations and patient consent

Approval for all procedures was obtained from the local ethic committee (‘Ethikkommission an der Medizinischen Fakultät der RWTH Aachen’, EK 192/20) and followed the Declaration of Helsinki. All individuals gave written informed consent before study participation. This study adheres to the relevant STROBE (Strengthening the Reporting of Observational Studies in Epidemiology) checklist, ensuring that all necessary components for reporting observational research are adequately addressed and documented.

### Procedures

#### Clinical measures

As previously described, we used standardized patient-reported outcome measures at all study timepoints including the Fatigue Scale for Motor and Cognitive Functions (FSMC), the Hospital Anxiety and Depression Scale (HADS-D), the Epworth Sleepiness Scale (ESS), and the Pittsburgh Sleep Quality Index (PSQI).^6,21^ Canadian Consensus Criteria for Myalgic Encephalomyelitis/Chronic Fatigue Syndrome (ME/CFS), the Bell Score (min. 0 = bedridden, max. 100 = no symptoms) for self-assessment of limitations^21^ and hand grip strength were only assessed in the last study visit. We used an electronic hand dynamometer to calculate the fatigue ratio (fmax/fmean; cut-off: >1.2 = decreased force), recovery ratio (fmean1/fmean2, cut-off: <0.9 = impaired muscle recovery) and hand grip strength as measures for general muscle strength, exertion and fatigability according to previously published studies.^22,23^

#### Neuropsychological assessments

The Montreal Cognitive Assessment (MoCA) served as a brief screening tool. The neuropsychological assessment included standardized measures for attention, information processing, and psychomotor speed (Trail Making Test-A, TMT-A; Alertness subtests of the Test of Attentional Performance, TAP), executive functions (phonemic and semantic verbal fluency; Digit Span backward; Trail Making Test-B, TMT-B; go/no-go task), language (Naming tasks and phonemic and semantic verbal fluency), visuospatial processing (Rey-Osterrieth Complex Figure copy, ROCFT), visual motor and processing speed (Symbol Digit Modalities Test, SDMT), as well as memory and learning (Complex Figure delayed recall; Digit Span forward; Verbal Learning and Memory Test, VLMT).^6,21^ Alternate test forms were used between study visits where applicable to avoid learning effects. Classifications of cognitive impairments were based on published normative data, which were adjusted for age, education and/or sex according to the specific test being used. Impairment was characterized as a performance falling below the 16th percentile rank or a *Z*-score between −2 and −1.5 and severe impairment as a percentile rank below 2 or a Z-score below −2.^24^

#### Fluid biomarker analysis

At each study visit blood samples were drawn and immediately analysed by our in-house laboratory at the UKA including c-reactive protein (CRP), interleukin 2 (IL-2), interleukin 6 (IL 6), interleukin 8 (IL 8), interleukin 10 (IL 10), interleukin II receptor (IL 2R), tumour necrosis factor alpha (TNF-α), antibodies against SARS Coronavirus 2 spike protein IgG and SARS Coronavirus 2 nucleoprotein IgG infection, D-dimers, fibrinogen, antithrombin III, ferritin, vitamin B12, folic acid and thyroid stimulating hormone. In parallel, a duplicate blood set was divided into 500-µl aliquots and stored at −80°C in the centralized biomaterial bank at the Faculty of Medicine at RWTH Aachen University and later used for measurement of NFL and GFAP at the Hertie Institute for Clinical Brain Research in Tübingen, Germany. To this end, samples were thawed on wet ice for 1 h. Afterwards, they were mixed for 30 s and centrifuged for 5 min at 10 000*×g* and 4°C. Measurements were performed on a single-molecule array platform (Simoa, HD-X analyzer; Quanterix) with commercially available assay kits (for further details, please see manufacturer’s instructions: https://www.quanterix.com/simoa-assay-kits/neurology-2-plex-b-nf-l-gfap-new/). All samples were measured in duplicates and blinded. Serum samples were 1:4 auto-diluted with Simoa sample diluent. Inter-assay variability was evaluated with specific human cerebrospinal fluid (CSF) samples as an internal reference. In total, in *n* = 4 of the serum samples, the coefficient of variation was >20%. Further, *n* = 2 single sample duplicates were below the limit of detection. No re-measurements have been performed.

#### MR imaging data acquisition

For all study participants, MRI data acquisition was performed using a Siemens PRISMA whole-body scanner with a 3T field strength (Siemens Healthlineers, Erlangen, Germany). High-resolution T1-weighted anatomical images and functional imaging data sequences were collected during resting-state conditions. Structural imaging was conducted using a T1-weighted sequence with an isotropic voxel resolution of 0.8 mm, an echo time (TE) of 2.36 ms, a repetition time (TR) of 2.4 s and a flip angle of 8°. Functional echo-planar imaging sequences were acquired with a matrix size of 64 × 64 × 36, capturing 205 volumes per session, with a voxel resolution of 3.1 × 3.1 × 3.6 mm, a TE of 30 ms, a TR of 2.21 s and a flip angle of 90°. During resting-state fMRI acquisition, the scanning environment was adjusted to reduce ambient light, and participants were instructed to keep their eyes open without focusing on specific thoughts, ensuring the capture of intrinsic brain activity in the absence of task-driven engagement or external stimuli.

#### Processing of imaging data

The multimodal imaging analysis followed a systematic processing framework, beginning with data organization in the Brain Imaging Data Structure format. T1-weighted anatomical scans and resting-state fMRI (rs-fMRI) scans then underwent a rigorous quality control (QC) assessment using MRIQC (v.23.1.0).^25^ Following automated QC, both anatomical and functional images were visually inspected for artifacts to ensure data integrity and suitability for further analysis. For functional data, motion-related exclusion criteria were systematically applied to minimize movement-induced artifacts. Participants were excluded if mean motion framewise displacement (mFD) exceeded 0.30 mm. A two-tiered QC approach was implemented to refine motion-related exclusions. Initially, a lenient threshold was used to identify potential motion outliers, defined as those with mFD greater than 0.55 mm, with exclusions applied as needed.^26^ Subsequently, a more stringent criterion was enforced, leading to participant exclusion if any of the following conditions were met: (i) mFD exceeded 0.30 mm, (ii) more than 20% of frames had an FD greater than 0.2 mm or (iii) any frame exhibited an FD exceeding 5 mm.^26^ Data from participants meeting these quality standards were further processed. For structural analysis, all participants were included. For functional analysis, the final dataset comprised 22 healthy controls at baseline, 19 at follow-up 1, and 19 at follow-up 2 and 47 PCC patients at baseline, 46 at follow-up 1, and 38 at follow-up 2 (Table 1).

**Table 1:** Imaging and clinical information

Visits	PCC patients			Healthy controls		
	Baseline	Follow-up 1	Follow-up 2	Baseline	Follow-up 1	Follow-up 2
Imaging						
T1, *n*	51	51	37	23	20	20
rs-fmri, *n*	47	46	38	22	19	19
Clinical information						
Sex female, *n* (%)*	33 (64.7)			13 (56.5)		
Age, years, mean (SD)*	47.3 (11.4)	48.4 (11.1)	49.9 (11.4)	35.2 (11.0)	36.2 (8.8)	37.2 (11.5)
Months since infection, mean (SD)	6.4 (3.6)	23.3 (4.5)	37.7 (5.3)	n.a.		
Hospitalization, *n* (%)	14 (27.5)			n.a.		
BMI, mean (SD)	26.6 (4.9)	27.1 (5.9)	27.2 (5.4)	n.a.		
Cardiovascular risk factors^a^, *n* (%)	18 (35.3)			n.a.		
Neurological comorbidities^b^, *n* (%)	14 (27.5)			0 (0)		
Psychiatric comorbidities^c^, *n* (%)	5 (9.8)			0 (0)		
Symptom questionnaires						
FSMC total, mean (SD)	64 (19.9)	62.1 (23.1)	60.1 (22.9)	n.a.		
HADS total, mean (SD)	12.3 (6.7)	10.2 (5.9)	9.2 (6.1)	n.a.		
MoCA, mean (SD)	26.7 (2.2)	25.8 (3.2)	26.8 (2.2)	n.a.		
ESS, mean (SD)	8.8 (5.6)	8.7 (4.9)	8.4 (4.7)	n.a.		
PSQI, mean (SD)	9.6 (4.3)	9.3 (4.5)	8.1 (3.7)	n.a.		
ME/CFS criteria fulfilled, *n* (%)			7 (15)	n.a.		
Bell Score, mean (SD)			66.2 (22.9)	n.a.		
Hand grip strength						
FmeanI, female/male, mean (SD),			21.4 (5.6)/33.6 (10.9)	n.a.		
Fmean2, female/male, mean (SD)			20.1(5.7)/31.5 (12.2)	n.a.		
Fmax, female/male mean, (SD)			25.6 (6.4)/38.2 (11.8)	n.a.		
Fatigue ratio, female/male, mean (SD)			1.2 (0.1)/1.1(0.1)	n.a.		
Recovery ratio, female/male, mean (SD)			1.1 (0.2)/1.2 (0.4)	n.a.		

Data are given as mean with standard deviation (SD) except for imaging, sex, hospitalization, cardiovascular risk factors and comorbidities, where counts are reported. ^a^including overweight (*n* = 12), arterial hypertension (*n* = 10), diabetes (*n* = 5), dyslipidemia (*n* = 3), ^b^including migraine (*n* = 7), mild head trauma (*n* = 2), transient ischemic attack (*n* = 1), benign brain tumour (*n* = 1), tension headache (*n* = 1), restless leg syndrome (*n* = 1), fibromyalgia (*n* = 1), ^c^including depression (*n* = 3), burn out (*n* = 2), posttraumatic stress disorder (*n* = 1).

Missing data: baseline FSMC *n* = 9, HADS *n* = 6, PSQI *n* = 16, ESS *n* = 11, MoCA *n* = 4; follow-up1 FSMC *n* = 3, HADS *n* = 1, PSQI *n* = 28, ESS *n* = 3, MoCA *n* = 0; follow-up 2 FSMC *n* = 1, HADS *n* = 0, PSQI *n* = 16, ESS *n* = 0, MoCA *n* = 0, Bell Score *n* = 1, ME/CFS *n* = 0. Cut-off values: FSMC total score: ≥43 mild fatigue, ≥53 moderate fatigue, ≥63 severe fatigue. HADS-D total score: ≤14 normal, 15–21 questionable, >21 increased. MoCA: <26 impaired global cognition, ≥26 normal global cognition. PSQI: ≤ 5 good sleepers, >5 poor sleepers. ESS: ≤ 10 average daytime sleepiness. >10 excessive daytime sleepiness. Bell Score: 0 = bedridden—100 = no symptoms. Hand grip strength for the age range 45–49 in females (18.6–32.4) and males (34.7–54.5); fatigue ratio (>1.2 = decreased force) and recovery ratio (<0.9 = impaired muscle recovery).

^*^No statistically significant differences were observed for age or sex between groups at any timepoint (*P* > 0.05).

#### Structural MRI data

Structural MRI data were segmented using FastSurfer (v.2.3.0), a deep learning-based pipeline, to extract structural metrics.^27^ This segmentation generated cortical thickness and volumetric measurements for 95 regions, covering cortical, subcortical and cerebellar structures. Specifically, subcortical volumes were obtained from the aseg (automatic segmentation) atlas, cortical thickness and volumes from the aseg + dkt (automatic segmentation + Desikan-Killiany-Tourville) atlas, and cerebellar volumes from the CerebNet atlas.^28,29^ To account for individual variability in brain size, all extracted measures were normalized by total intracranial volume, which was obtained from the aseg atlas output. These structural metrics were then analysed statistically to assess group differences and structural associations.

#### Functional MRI data

The rs-fMRI data were preprocessed using the fMRIPrep pipeline (v23.1.3) following our previously published protocol^6^ (Supplementary Text S1). After preprocessing, denoising and spatial smoothing were conducted using the CONN toolbox (version conn22v2407) in MATLAB.^30^ Confounds identified in the preprocessing step were regressed out, including five principal components each from white matter and CSF, session effects and their first-order derivatives (two components) and polynomial trends (three components). BOLD signals were band-pass filtered (0.008–0.09 Hz) to isolate functionally relevant neural activity. Spatial smoothing was applied with an 8 mm full-width at half maximum Gaussian kernel to enhance signal-to-noise ratio, comparability and data normality.^30^ To examine both regional neuronal activity and functional connectivity, analyses were performed using the CONN toolbox (version conn22v2407) in MATLAB, with custom scripts automating the workflow.^31^ Regional spontaneous neural activity was quantified via amplitude of low-frequency fluctuations (ALFF). ALFF identified activated regions were then used for ROI-to-ROI connectivity analysis based on the Harvard-Oxford atlas.^32^ This integrative approach allowed a detailed assessment of both localized neuronal dynamics and large-scale functional network interactions, identifying region-specific changes and broader network disruptions.

### Statistical analysis

Our study employed a longitudinal analytical approach. We investigated structural, functional, clinical and fluid data in PCC patients, alongside structural and functional neuroimaging data in healthy controls (Supplementary Text S2). Data were assessed using linear mixed-effects (LME) models to identify significant effects and connectivity changes. LME models effectively accounted for repeated measurements in longitudinal data and accommodated missing values (Supplementary Text S3).^33^ Age and sex were included as covariates to control for potential confounding. Additionally, a separate analysis was conducted after excluding PCC patients who had fully recovered at follow-up 2. Statistical significance was set at *P* < 0.05, corrected for multiple comparisons using the Benjamini–Hochberg false discovery rate (p-FDR). Structural analyses were conducted in R (v4.3.1), while functional connectivity analyses were performed at the group level using the CONN toolbox in MATLAB (vR2022a).^31^ Connectivity measures were clustered based on anatomical and functional similarity using hierarchical clustering, allowing meaningful comparisons within and between networks. Variance homogeneity was assessed via *F*-tests, and effect sizes (small: 0.2, medium: 0.5, large: 0.8)^34^ were calculated to quantify the magnitude of significant findings. Finally, Spearman’s correlation analyses,^33^ corrected for multiple comparisons using FDR, explored associations among structural, functional, clinical, neuropsychological and fluid measures.

## Results

### Demographic and clinical characteristics

**Figure 1:**
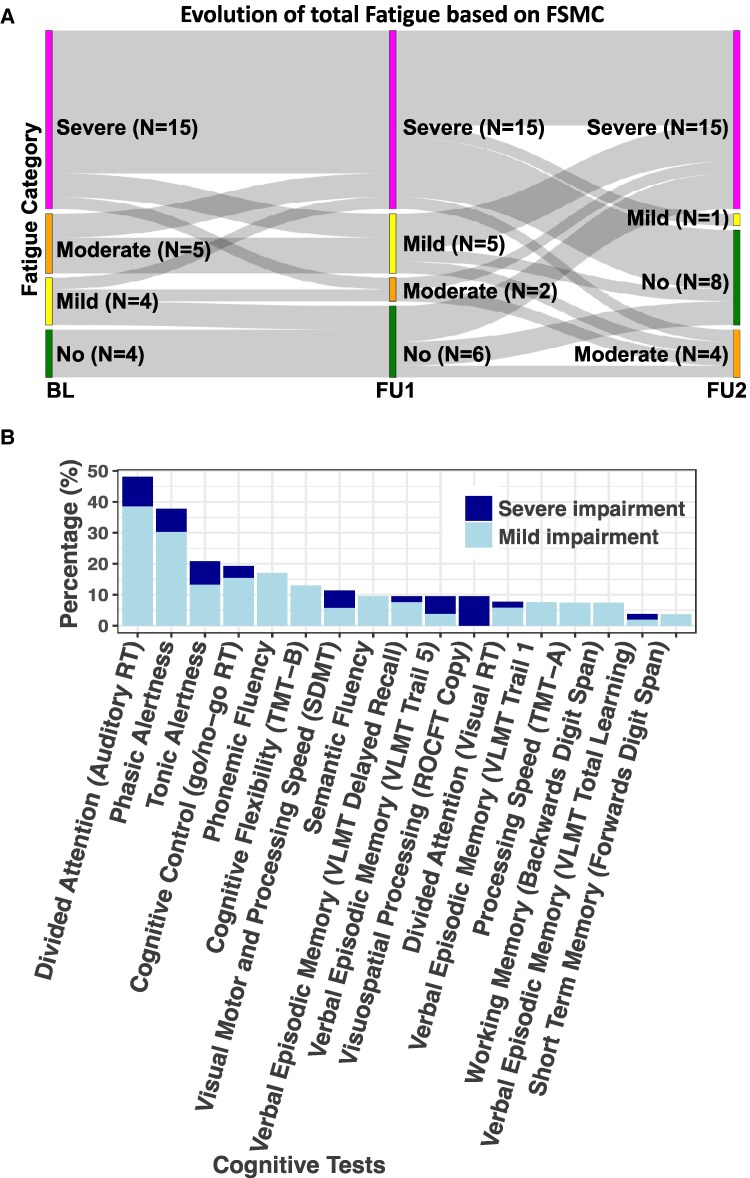
**Evolution of fatigue and neuropsychological assessment in post-COVID patients over three years.** (**A**) Sankey-diagram indicates the self-assessed overall fatigue trajectories based on the Fatigue Scale for Motor and Cognitive Functions (FSMC). Only data where the FSMC was available at all three time points (*N* = 28) are shown. (**B**) Stacked bar plots showing the percentage of patients (*N* = 38) for mild and severe cognitive performance 38 months after infection adjusted for age, sex and education, where applicable. Abbreviations*: RT*, response time; *TMT*, Trail Making Test; *SDMT*, Symbol Digit Modalities Test; *VLMT*, Verbal Learning and Memory Test; *ROCFT*, Rey–Osterrieth Complex Figure copy.

Demographic and clinical information on PCC patients and healthy controls are shown in Table 1. Forty-six participants withdrew for various reasons (unresponsive/unreliable *n* = 20, recovered *n* = 3, death *n* = 1, pregnancy *n* = 1, too ill to participate due to PCC *n* = 6, other reasons *n* = 15). Prior to study inclusion, 14 (27%) patients had been hospitalized during acute COVID-19 including 6 (12%) patients with treatment on an intensive care unit. Patient-reported outcomes showed an on average improved but persistent, moderate fatigue, although a subgroup worsened over time (Fig. 1A). At each study timepoint, there were no signs for affective symptoms, global cognitive function was normal and the amount of daytime sleepiness was rated as average, although the PSQI indicated continuously poor sleep quality. The majority of patients reported an improvement of symptoms, among which fatigue, cognitive complaints and a reduced functional capacity were still most commonly reported after three years (Supplementary Fig. 1). The mean Bell Score was 66 corresponding to mild symptoms at rest with noticeable limitations in daily activities and an overall functional status of about 70–90% of normal. Seven (15%) patients formally fulfilled ME/CFS criteria at the final study visit. Based on a Bell Score of ≥90, 12 (24%) patients have claimed to be fully recovered. According to published reference values derived from healthy controls, measures of hand grip strength did not indicate increased muscle weakness, fatiguability or impaired muscle recovery.^22,23^

### Neuropsychological assessments

Cognitive performance three years after acute COVID-19 is shown in Fig. 1B. More than 80% of PCC patients demonstrated cognitive performance within the normal range across the majority of assessed domains. Impairments were observed most frequently in the domain of divided attention, with 48% of patients showing at least mild deficits. Phasic alertness was affected in 38% of patients, and tonic alertness in 21%. Lowest proportions of impairment were observed for verbal episodic memory and short-term memory, where most patients (>95%) performed within normal ranges.

### Fluid biomarkers

**Figure 2:**
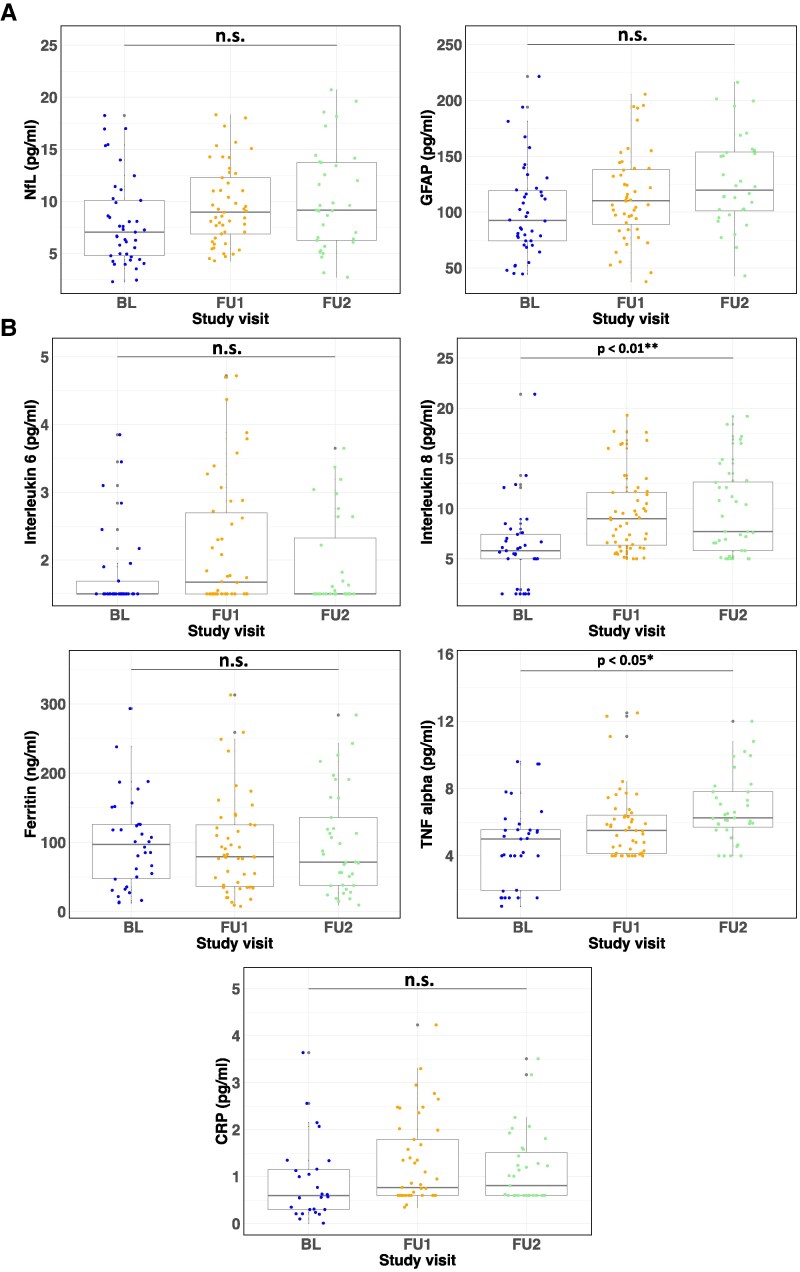
**Evolution of neurodestruction and inflammatory markers in post-COVID patients over three years.** Boxplots showing the temporal evolution of fluid markers for baseline (BL = blue, 6 months), follow-up 1 (FU1 = orange, 12 months) and follow-up 2 (FU2 = green, 38 months) and respective sample size (**N**) of each timepoint (BL/FU1/FU2) after acute COVID-19. (**A**) Neurofilament light (NfL) (reference range according to mean age <15 pg/ml, *N* = 41/46/33) and glial fibrillary acidic protein (GFAP) (reference range according to mean age <242 pg/ml, *N* = 42/45/32). (**B**) Inflammatory markers associated with postinfectious fatigue and PCC. Interleukin 6 (reference range <7.0 pg/ml, *N* = 33/42/32), interleukin 8 (reference range <62 pg/ml, *n* = 33/46/36), tumour necrosis factor (TNF) alpha (reference range <8.1 pg/ml, *N* = 33/45/35), ferritin (reference range 15.0–150 ng/ml, *N* = 34/44/37) and C-reactive protein (CRP) (<5.0 pg/ml, *N* = 26/42/35). Outliers that were 1.5 times the interquartile range were excluded to enhance visualization. Each data point indicates the measurement from one patient. Statistical analysis was performed using LME models, including age and sex as covariates. Multiple comparisons were corrected using FDR correction, with significance set at *P* < 0.05.

Information on all fluid biomarkers is shown in Table 2. Laboratory tests on thyroid functioning, blood sugar, coagulation, vitamin B12 and folic acid, to exclude contributing factors of symptoms, showed no abnormalities. Evolution of neuronal injury and inflammatory markers previously associated with postinfectious fatigue and PCC are shown in Fig. 2A and B. There was a significant increase of IL 2 (*P* < 0.001), IL 8 (*P* < 0.01), IL 10 (*P* < 0.001) and TNF alpha (*P* < 0.05) over time (Supplementary Table 1), but all laboratory parameters were within the normal range. Considering the mean age of the cohort, NfL and GFAP were also within published normative reference values.^35,36^

**Table 2:** Fluid markers in PCC patients

Visits	PCC patients		
	Baseline	Follow-up 1	Follow-up 2
Fluid markers			
IL 2, pg/ml, mean (SD)	1.3 (0.9)	3.0 (2.7)	2.6 (0.0)
IL 6, pg/ml, mean (SD)	2.0 (1.1)	2.7 (1.8)	3.0 (2.8)
IL 10, pg/ml, mean (SD)	1.4 (0.6)	3.5 (4.3)	6.9 (10.9)
IL 8, pg/ml, mean (SD)	7.7 (9.4)	11.1 (8.1)	11.8 (8.4)
IL 2-R, U/ml, mean (SD)	269.5 (176.8)	335.7 (105.6)	386.1 (149.7)
TNF alpha, pg/ml, mean (SD)	5.4 (3.9)	7.8 (12.0)	8.9 (9.1)
Ferritin, ng/ml, mean (SD)	166.8 (395.9)	136.1 (148.9)	124.8 (120.4)
CRP, pg/ml, mean (SD)	2.0 (3.7)	13.2 (73.6)	2.0 (2.6)
D-Dimers, µg/l, mean (SD)	302.6 (219.4)	322.3 (213.3)	314.9 (177.7)
Fibrinogen, mg/dl, mean (SD)	286.4 (52.8)	301.1 (53.8)	302.7 (49.9)
Antithrombin III, %, mean (SD)	106.6 (10.6)	103.0 (10.8)	102.8 (10.4)
Folic acid, ng/ml, mean (SD)	7.9 (3.7)	8.3 (4.3)	9.2 (5.0)
Vitamin B12, pg/ml, mean (SD)	485.5 (298.1)	471.1 (157.2)	490.4 (166.3)
Spikeprotein IgG, BAU/ml, mean (SD)	261.2 (165.0)	389.0 (271.5)	2080.0 (0.0)
Nucleoprotein IgG, AU/ml, mean (SD)	55.1 (54.1)	41.0 (45.0)	56.0 (41.0)
NfL, pg/ml, mean (SD)	10.8 (16.2)	10.2 (4.4)	10.2 (4.9)
GFAP, pg/ml, mean (SD)	162.1 (397.8)	120.8 (52.0)	133.5 (51.5)

Data are given as mean with standard deviation (SD). Reference range: IL 2 (<5.0 pg/ml), IL 6 (<7.0 pg/ml), IL 10 (<9.1 pg/ml), IL 8 (<62 pg/ml), IL 2-R (223–710 U/ml), TNF alpha (<8.1 pg/ml), ferritin (15.0–150 ng/ml), CPR (<5.0 pg/ml), D-Dimers (<500 µg/l), fibrinogen (238–498 mg/dl), antithrombin III (80–120%), folic acid (>4.4 pg/ml), vitamin B12 (197–771 pg/ml), spikeprotein (negative <33.8, positive ≥ 33.8 BAU/ml), nucleoprotein (negative <20, positive ≥24 AU/ml), NfL (according to mean age <15 pg/ml), GFAP (according to mean age <242 pg/ml).

### Structural brain changes

**Figure 3:**
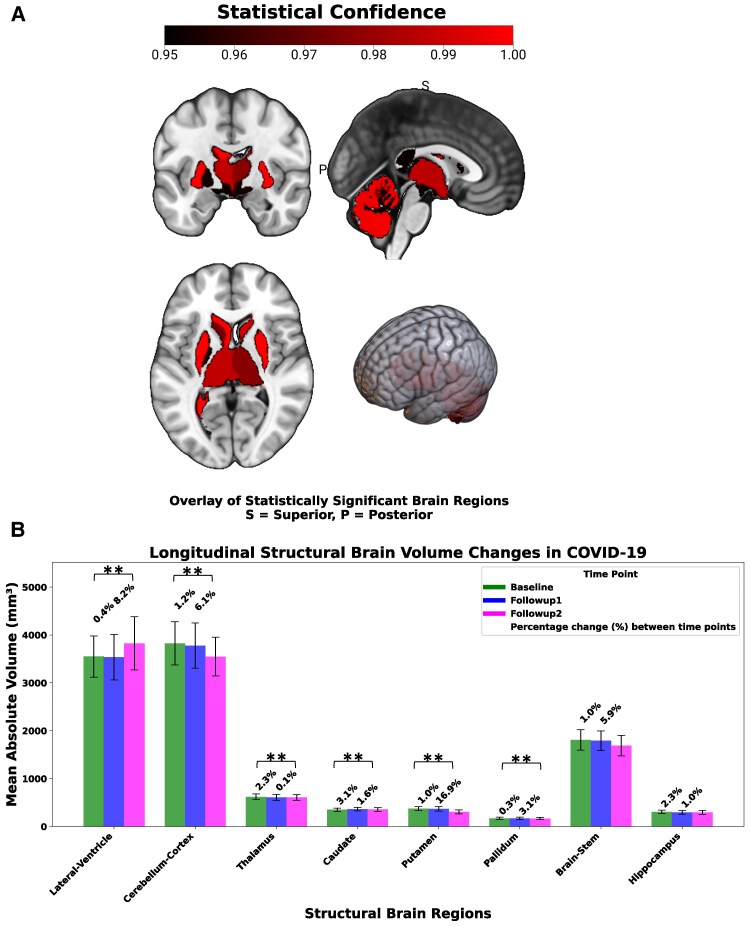
**Longitudinal structural brain changes in post-COVID patients over three years.** The structural dataset included *N* = 51 PCC patients (BL: 51, FU1: 51, FU2: 37) and *N* = 23 healthy controls (BL: 23, FU1: 20, FU2: 20). (**A**) Brain regions showing statistically significant longitudinal volumetric changes in PCC patients, derived using LME models. Age and sex were included as covariates. The overlay represents 1 − *P*-values (range: 0.95 to 1.00), with a threshold of p-FDR < 0.05, indicating statistical confidence changes over time. These results are displayed as brain overlays from lateral, medial and dorsal perspectives. (**B**) Bar plots showing the percentage change in volumetric scores for each statistically significant brain region across the three timepoints (BL, FU1, FU2). Each data point represents the mean percentage volume for that region in PCC patients at each timepoint. Error bars indicate standard deviation. Statistical analysis was performed using LME models, corrected for multiple comparisons using the FDR approach. Asterisks indicate statistically significant changes (*P < 0.05 and **P < 0.01).

All statistically significant structural brain changes including effect sizes are shown in Fig. 3A and Supplementary Table 2. Across all three study timepoints we identified significantly changed brain regions within PCC patients, whereas healthy controls revealed no significant structural alterations. Amongst others, in PCC patients a volume decrease was detected for the left pallidum (3.4%, *P* < 0.05) and in both hemispheres for the thalamus (2.4%, *P* < 0.05), the putamen (17.9%, *P* < 0.001), the caudate nucleus (4.7%, left *P* < 0.05; right *P* < 0.01) and parts of the cerebellar cortex (7.3%, left *P* < 0.05; right *P* < 0.001) (Fig. 3B and Supplementary Table 2). Both lateral ventricles (8.6%, *P* < 0.001) and the third ventricle (5.9%, *P* < 0.05) showed an increase in volume over time. Notably, most of these changes were also detectable after removing all fully recovered individuals from the analysis. The brainstem and the choroid plexus showed no significant structural alterations.

### ALFF and functional connectivity

**Figure 4:**
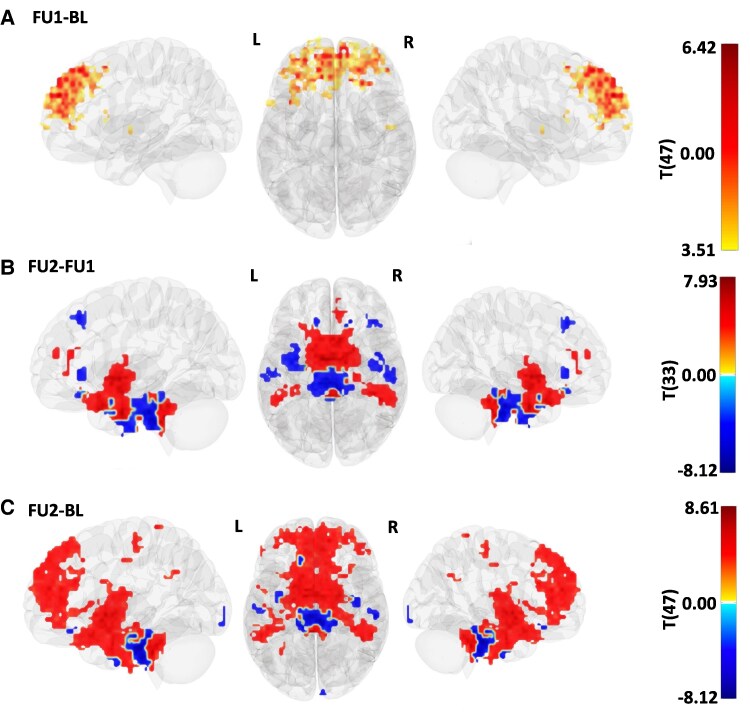
**Longitudinal ALFF brain changes in post-COVID patients over three years.** Shown are all brain regions with significant changes in neuronal activation from (**A**) baseline to follow-up 1 (FU1 − BL), (**B**) follow-up 1 to follow-up 2 (FU2 − FU1), and (**C**) baseline to follow-up 2 (FU2 − BL) in post-COVID patients from lateral and dorsal perspectives. Red colour indicates increased and blue colour decreased activation. Statistical analysis was performed using LME models with age and sex included as covariates. Multiple comparisons were corrected using the FDR approach. The initial cohort included *N* = 51 PCC patients and *N* = 23 healthy controls; after quality control, the final functional dataset comprised *N* = 47 PCC patients (BL: 47, FU1: 46, FU2: 38) and *N* = 22 healthy controls at baseline, 19 at FU1, and 19 at FU2. Each voxel represents the statistical confidence of longitudinal changes in ALFF over time.

All brain regions with significantly altered neuronal activation from baseline to the final follow-up visit are shown in Fig. 4 and Supplementary Table 3. The ALFF analysis across all three timepoints revealed a dynamic pattern of activation: two years after infection, the observed exclusive overactivation in frontal regions at baseline (Fig. 4A) turned into a more balanced pattern of regions showing both increased and decreased neuronal activation in the brainstem, cerebellum, parahippocampal gyrus and temporal regions (Fig. 4B). Three years after acute COVID-19 patients with PCC revealed a predominant pattern of increased activation (Fig. 4C). For both hemispheres, these regions included the thalamus, caudate nucleus, pallidum, the putamen and the hippocampus. The brainstem showed decreased activation.

We performed an additional ROI analysis three years after infection, setting the abnormal structural and activity regions as seeds (Supplementary Table 4). We found an overall decreased functional connectivity, except for the brainstem and the cerebellum crus 2, which exhibited an increased functional connectivity.

### Correlations of neuropsychiatric assessments and fluid markers with brain changes

**Figure 5:**
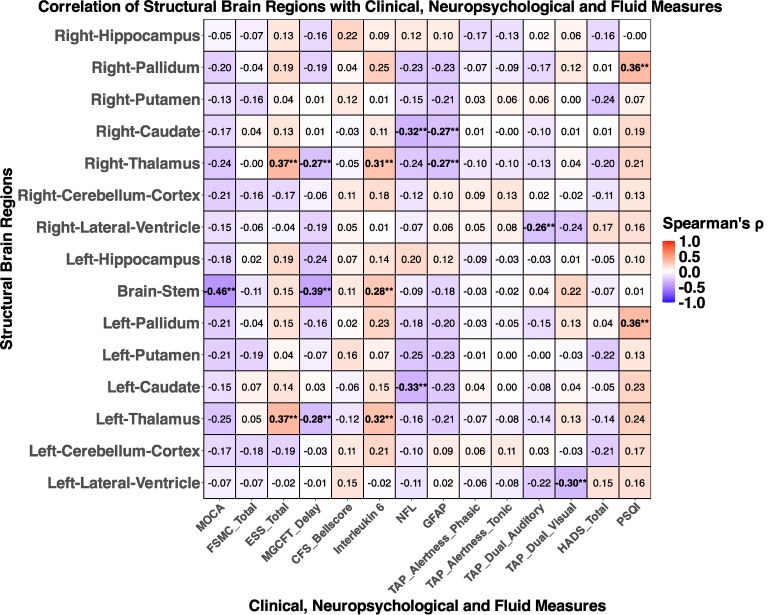
**Correlation heatmap illustrating the associations between clinical, neuropsychological and fluid markers with structural brain changes in post-COVID patients after three years.** Spearman’s rank correlation was used to assess associations. Statistical significance for correlation measures was determined using FDR correction (*P* < 0.05). Asterisks indicate statistically significant correlations (*P < 0.05 and **P < 0.01). Each cell in the heatmap represents a pairwise correlation between a marker and a brain region. Analyses were performed using data from PCC patients with available three-year follow-up data (*N* = 37). Abbreviations*: MoCA*, Montreal Cognitive Assessment; *FSMC*, Fatigue Scale for Motor and Cognitive Functions; *ESS*, Epworth Sleepiness Scale; *MGCFT*, Modified copy figure test; *CFS*, Chronic Fatigue Syndrome, *NFL*, Neurofilament light, *GFAP*, Glial Fibrillary Acidic Protein; *TAP*, Test of Attentional Performance; *HADS*, Hospital Anxiety and Depression Scale; *PSQI*, Pittsburgh Sleep Quality Index.

**Figure 6:**
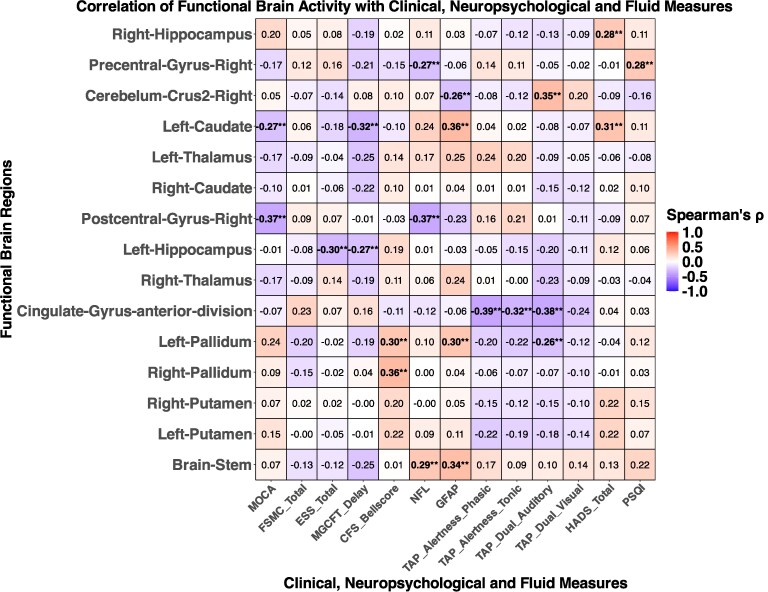
**Correlation heatmap illustrating the associations between clinical, neuropsychological and fluid markers with ALFF brain changes in post-COVID patients after three years.** (Spearman’s rank correlation was used to assess associations. Statistical significance for correlation measures was determined using FDR correction (*P* < 0.05). Asterisks indicate statistically significant correlations (*P < 0.05 and **P < 0.01). Each cell in the heatmap represents a pairwise correlation between a marker and a brain region. Analyses were performed using data from PCC patients with available three-year follow-up data (*N* = 38). Abbreviations*: MoCA,* Montreal Cognitive Assessment; *FSMC,* Fatigue Scale for Motor and Cognitive Functions; *ESS,* Epworth Sleepiness Scale; *MGCFT* copy figure test; *CFS,* Chronic Fatigue Syndrome; *NFL,* Neurofilament light; *GFAP,* Glial Fibrillary Acidic Protein; *TAP,* Test of Attentional Performance; *HADS,* Hospital Anxiety and Depression Scale; *PSQI,* Pittsburgh Sleep Quality Index.

Neuropsychiatric and fluid marker correlations with structural and functional brain changes are shown in Figs 5 and 6. Structurally, measures of non-verbal recall were negatively correlated with the volumetric scores of the thalamus (left: *r* = −0.28; right: *r* = −0.27) and the brainstem (*r* = −0.39). Daytime sleepiness according to the ESS score (bilateral: *r* = 0.37) was positively associated with volumetric scores of the thalamus and sleep quality according to the PSQI with the volume of the pallidum (bilateral: *r* = 0.36). On a functional level, measures of attention were negatively correlated with the volumetric score of the cingulate gyrus. Non-verbal recall was negatively correlated with the volume of the left caudate nucleus (*r* = −0.32) and the left hippocampus (*r* = −0.27). Correlations of structural and functional brain changes are shown in Supplementary Fig. 2. Notably, the strong associations between structural and functional changes we observed for the pallidum and putamen after two years were no longer detectable three years after acute COVID-19.

## Discussion

In this longitudinal study, we aimed to identify signs and associated factors of brain recovery in PCC by tracking neuropsychiatric, fluid biomarker and imaging trajectories three years after acute COVID-19.

We observed a slow clinical improvement, alongside structural changes and widespread neuronal activation in periventricular regions without signs of neurodegeneration or systemic inflammation, suggesting an overall beginning restoration of brain function.

Three years after infection, the average FSMC score only dropped by four points in total, still corresponding to moderate fatigue. Limitations of the FSMC in detecting fatigue frequency and higher cut-off values have previously been discussed.^37^ However, there were no signs of increased muscular fatigue or impaired muscular recovery and the overall functional status was reported to range from 70% to 90% of normal on average. In addition, the majority of patients reported an improvement of symptoms and almost one quarter claimed to have fully recovered. Only 15% of patients met the criteria for ME/CFS, a severe neuroinfectious syndrome characterized by post-exertional malaise, which is substantially less than previous reports of more than 50%^23^ and 20%.^6^ Nevertheless, in the context of the COVID-19 pandemic, this persistent ME/CFS-like subgroup warrants significant clinical attention and research investment. Current understanding of the syndrome remains limited, increasing the risk of misdiagnosis and long-term chronicity. Targeted clinical interventions such as educating patients on energy management strategies, implementing symptom-specific pharmacologic treatments, and prioritizing inclusion in dedicated research trials, are essential to improve care and guide the development of effective therapies moving forward.

Regarding cognitive functioning, divided attention and alertness were still the most frequently affected domains, while the majority of patients showed no clinically relevant impairment in cognitive assessments as previously reported.^37^

Considering brain changes, we found a significant volume increase of ∼8% in both lateral ventricles and of ∼6% in the third ventricle, also previously reported in our two-year follow-up study.^6^ While there was no significant enlargement of the lateral ventricles in our healthy control cohort over time, it is considered a typical feature of brain aging^38^ that occurs at a rate of 2.23% per year.^39^ This points to an overall moderate effect in PCC considering that a cumulative increase of approximately 6.7% would be expected over three years. An explanation for ventricle increase could be an enlargement of the choroid plexus due to inflammation-triggered swelling during the acute phase but also in the long term, which was found in a study on 129 PCC patients 15 months after infection to moderate gray matter volume reduction and cognitive performance.^3^ The authors proposed that either choroid plexus vulnerability during the acute infection and/or postacute damage from immune activation could lead to the entry of immune cells and cytokines, fostering neuroinflammation and brain damage. On a molecular level, it was shown that SARS-CoV-2 neurotropism predominates in choroid plexus epithelium,^40^ possibly explaining the blood-brain barrier disruption found during acute infection and in PCC patients suffering from *brain fog*.^4^ Strikingly, choroid plexus enlargement seems to be a SARS-CoV-2-specific effect rather than a general non-COVID-19 infection-triggered effect in patients with encephalopathy.^41^

Although there was a trend to increase, we did not find a statistically significant choroid plexus enlargement, indicating the need for more refined segmentation approaches of this structure.^42^ However, we found a significant bilateral volumetric decrease in structures located in close proximity to the ventricular system. These structures included the thalamus, caudate nucleus and globus pallidus, adjacent to the lateral ventricles and to the third ventricle as well as the cerebellum forming the back of the fourth ventricle. The thalamic volume reduction was also associated with impaired memory function and daytime sleepiness. This is in line with a previous study, which found aberrant fractional anisotropy of the thalamus and shape deformations and decreased volumes of the left thalamus, putamen and pallidum correlating with measures of fatigue, sleepiness and verbal memory in PCC patients 7.5 months after acute COVID-19.^7^

Given that the lateral ventricles contain the largest part of the choroid plexus, and hence are more vulnerable to profound structural alterations in adjacent areas, we assumed that other regions, such as the brainstem recover earlier. In a previous cross-sectional study, we found that volume loss of the brainstem was still detectable up to two years post COVID-19 but its association to fatigue dissolved, pointing to non-neuronal factors underlying this symptom.^6^ Three years after COVID-19, brainstem reduction was no longer detectable.

To map the temporal evolution of brain recovery regarding the effects of structural changes on functional compensation, we included analyses of ALFF and functional connectivity in our study. Enhanced neuronal activation and increased connectivity alongside volume loss suggest efficient brain compensation within recovering regions. In contrast, increased activation with decreased connectivity points to earlier stages of brain recovery with yet insufficient compensation.

The brainstem showed decreased neuronal activity but increased functional connectivity pointing to an efficient compensation following initial volume loss in earlier assessments.^6^ In contrast, we found that the volume decreases we detected in the thalamus and the basal ganglia were accompanied by significantly increased neuronal activation, whereas local functional connectivity was decreased in these brain areas. These findings suggest early stages of brain recovery with primarily compensatory mechanisms in the basal ganglia and thalamus, although the observation of continued volume reduction warrants careful interpretation. While some studies report measurable brain recovery 6 to 10 months post-infection,^43,44^ others document persistent structural alterations up to two years later.^45,46^ With respect to the gradual clinical improvement, we interpret our findings as indicative of an initial step towards recovery with the brainstem exhibiting the first signs of structural stability and efficient network compensation, followed by the basal ganglia and the thalamus.

To investigate whether ongoing inflammation and neuronal injury account for the brain alterations we found, we tracked blood levels of cytokines, NfL and GFAP over a time span of three years. Existing literature yields varying results on these markers in PCC patients and there is no such long-term study up to date. Similar to our findings, most studies did not find signs of long-term neurodegeneration or association with structural abnormalities and elevated cytokines^17^ even in PCC patients with cognitive impairment.^15,16^ This indicates that the longitudinal structural decrease is a result of cellular shrinking rather than neurodegeneration. However, one study on 84 PCC patients found imaging changes in selective hippocampal subfields to be associated with altered GFAP and NfL levels and cognitive impairment in attention and memory.^18^ Moreover, a recent biobank study from the UK provided further evidence that mild-to-moderate SARS-CoV-2 infection may initiate or accelerate brain β-amyloid pathology, although neuronal injury markers were not elevated.^47^ While NfL and GFAP may not be suitable biomarkers for PCC, this emphasizes the importance of viral pathogens as relevant triggers or contributors to neurodegenerative diseases at least in a subset of patients.

Regarding inflammation, persistent inflammatory markers including IL-6, IL8, TNF alpha and CRP, have previously been associated with postinfectious fatigue and PCC^13,23,48^ and immune dysregulation is considered to be one possible pathomechanism driving post-COVID symptoms.^11^

For instance, Talla *et al*. demonstrated that persistent serum protein signatures, especially those associated with TNF characterize an inflammatory subtype of long COVID.^49^ The significant increase of TNF alpha in our study may reflect this finding and we also found some associations of neuronal injury markers and interleukins between altered brain regions. However, the clinical relevance is questionable considering that all inflammatory and neuronal injury markers were within the normal range.

The lack of detectable inflammatory markers indicates that the inflammatory response and subsequent effects are largely confined to the acute phase of COVID-19. Importantly, despite the distinct severity of acute COVID-19 among PCC patients, also present in our study cohort, a two-year follow-up study found no differences in post-COVID symptoms between hospitalized and non-hospitalized patients.^50^ Correspondingly, our previous study revealed similar brain changes in both groups, though with greater prominence in hospitalized patients.^6^ Thus, addressing acute inflammation by vaccination strategies and antiviral drugs in middle-aged and younger patients who often manage potentially severe infections at home and are classified as mild cases could potentially improve recovery and reduce the risk of PCC.

Our study has some limitations. First, the major limitation is the absence of an asymptomatic control group after SARS-CoV-2 infection, which was not available within our study. Rau and colleagues reported that brain changes were detected in both symptomatic and asymptomatic individuals challenging the validity of pre-pandemic healthy control data that most neuroimaging studies including ours, use for comparison.^51^ In addition, information on pre-existing symptoms and cognitive functioning in PCC patients were not available, potentially confounding our results. Given it is a monocentric study, potential selection bias cannot be ruled out. Due to the longitudinal design of our study, we encountered loss to follow-up, limiting the generalizability of our findings. Lastly, given the lack of repeated neuropsychological testing within the control group, we did not account for retest-effects.

The major strength of our study lies in the integrated analysis of multimodal, state-of-the-art imaging techniques alongside fluid biomarker analyses of neurodegeneration and inflammation, enabling us to draw meaningful conclusions about the nature of the observed changes. In addition, this is, to the best of our knowledge, the longest follow-up neuroimaging study on PCC patients providing valuable longitudinal insights on COVID-19-associated brain changes.

In conclusion, clinical improvement is slow but evident three years following acute COVID-19, manifesting in gradual improvement of fatigue and cognition and return to previous functional status without any signs of ongoing systemic inflammation. On a brain level, recovery is reflected by efficient neuronal recruitment and increased activation within the basal ganglia as well as structural stabilization alongside increased connectivity, starting in the brainstem first, with no evidence of neurodegeneration. These findings underscore the complex yet positive trajectory of recovery in PCC patients over the long term.

## Supplementary Material

fcaf366_Supplementary_Data

## Data Availability

Deidentified imaging, clinical and neuropsychological data are available on request from the corresponding author. All scripts used for data preprocessing, statistical modeling and figure generation are publicly available at https://github.com/ravidadsenaiitm/LNC_FU_Codes.
