# Supplementary material for: Evidence of clinical and brain recovery in post-COVID-19 condition: a three-year follow-up study
Source: Brain Commun. 2025 Sep 23;7(5):fcaf366. doi: 10.1093/braincomms/fcaf366 (PMC12508649; doi:10.1093/braincomms/fcaf366)
Supplement: fcaf366_Supplementary_Data [file fcaf366_supplementary_data.docx]

**SUPPLEMENTARY MATERIAL**

Table of content

[Supplementary Text S1 Detailed information of fmriprep preprocessing pipeline 2](#_Toc209121473)

[**Structural data preprocessing** 2](#_Toc209121474)

[**Functional data preprocessing** 2](#_Toc209121475)

[Supplementary Text S2 Detailed statistical analysis 3](#_Toc209121476)

[Supplementary Text S3 Detailed information on handling and removing data 4](#_Toc209121477)

[Supplementary Table 1 Longitudinal analysis of clinical and fluid biomarkers from baseline to follow-up 2 in post-COVID patients 4](#_Toc209121478)

[Supplementary Table 2 Statistically Significant longitudinal changes in structural brain regions in post-COVID patients from baseline to follow-up 2 4](#_Toc209121479)

[Supplementary Table 3 Statistically Significant longitudinal clusters identified by ALFF analysis in post-COVID patients from baseline to follow-up 2 5](#_Toc209121480)

[Supplementary Table 4 ROI Analysis of longitudinal functional connectivity changes in post-COVID patients 8](#_Toc209121481)

[Supplementary Fig. 1 Stacked barplot showing symptom progression from baseline to follow-up 2 in post-COVID patients 9](#_Toc209121482)

[Supplementary Fig. 2 Correlation heatmap of structural and ALFF brain changes in post-COVID patients. 10](#_Toc209121483)

[References 10](#_Toc209121484)

#

# **Supplementary Text S1 Detailed information of fmriprep preprocessing pipeline**

## **Structural data preprocessing**

A total of 1 T1-weighted (T1w) images were found within the input BIDS dataset. The T1-weighted (T1w) image was corrected for intensity non-uniformity (INU) with N4BiasFieldCorrection^1^, distributed with antsRegistration ANTs (v.23.2.08) ^2^, and used as T1w-reference throughout the workflow. The T1w-reference was then skull-stripped with a Nipype implementation of the antsBrainExtraction.sh workflow (from ANTs), using OASIS30ANTs as target template. A total of 1 T1-weighted (T1w) images were found within the input BIDS dataset. The T1-weighted (T1w) image was corrected for intensity non-uniformity (INU) with N4BiasFieldCorrection ^1^, distributed with ANTs (version unknown)^2^, (RRID:SCR_004757), and used as T1w-reference throughout the workflow. The T1w-reference was then skull-stripped with a Nipype implementation of the antsBrainExtraction.sh workflow (from ANTs), using OASIS30ANTs as target template. Brain tissue segmentation of cerebrospinal fluid (CSF), white-matter (WM) and gray-matter (GM) was performed on the brain-extracted T1w using fast (FSL (v.6.0.6.5).^3^ Brain surfaces were reconstructed using recon-all (FreeSurfer 7.3.2, RRID:SCR_001847)^4^, and the brain mask estimated previously was refined with a custom variation of the method to reconcile ANTs-derived and FreeSurfer-derived segmentations of the cortical gray-matter of Mindboggle.^5^ Volume-based spatial normalization to one standard space (MNI152NLin2009cAsym) was performed through nonlinear registration with ANTs, using brain-extracted versions of both T1w reference and the T1w template. The following template were selected for spatial normalization and accessed with TemplateFlow (23.0.0)^6^: ICBM 152 Nonlinear Asymmetrical template version 2009c^7^ [TemplateFlow ID: MNI152NLin2009cAsym].

## **Functional data preprocessing**

For each functional BOLD run (across all sessions), the following preprocessing steps were implemented. First, a reference volume and its skull-stripped version were generated using a custom methodology of fMRIPrep. Head-motion parameters with respect to the BOLD reference (transformation matrices, and six corresponding rotation and translation parameters) are estimated before any spatiotemporal filtering using mcflirt (FSL)^8^. The estimated fieldmap was then aligned with rigid-registration to the target EPI (echo-planar imaging) reference run. The field coefficients were mapped on to the reference EPI using the transform. BOLD runs were slice-time corrected to 1.06s (0.5 of slice acquisition range 0s-2.13s) using 3dTshift from AFNI^9^ (RRID:SCR_005927). The BOLD reference was then co-registered to the T1w reference using bbregister (FreeSurfer) which implements boundary-based registration.^10^ Co-registration was configured with six degrees of freedom. Several confounding time-series were calculated based on the preprocessed BOLD: framewise displacement (FD), DVARS and three region-wise global signals. FD was computed using two formulations following Power (absolute sum of relative motions^11^) and Jenkinson (relative root mean square displacement between affines ^8^). FD and DVARS are calculated for each functional run, both using their implementations in Nipype (following the definitions by Power et al. 2014).^11^ The three global signals are extracted within the CSF, the WM, and the whole-brain masks. Additionally, a set of physiological regressors were extracted to allow for component-based noise correction (CompCor).^12^ Principal components are estimated after high-pass filtering the preprocessed BOLD time-series (using a discrete cosine filter with 128s cut-off) for the two CompCor variants: temporal (tCompCor) and anatomical (aCompCor). tCompCor components are then calculated from the top 2% variable voxels within the brain mask. For aCompCor, three probabilistic masks (CSF, WM and combined CSF+WM) are generated in anatomical space. The implementation differs from that of Behzadi et al. in that instead of eroding the masks by 2 pixels on BOLD space, a mask of pixels that likely contain a volume fraction of GM is subtracted from the aCompCor masks.^12^ This mask is obtained by dilating a GM mask extracted from the FreeSurfer’s aseg segmentation, and it ensures components are not extracted from voxels containing a minimal fraction of GM. Finally, these masks are resampled into BOLD space and binarized by thresholding at 0.99 (as in the original implementation). Components are also calculated separately within the WM and CSF masks. For each CompCor decomposition, the k components with the largest singular values are retained, such that the retained components’ time series are sufficient to explain 50 percent of variance across the nuisance mask (CSF, WM, combined, or temporal). The remaining components are dropped from consideration. The head-motion estimates calculated in the correction step were also placed within the corresponding confounds file. The confound time series derived from head motion estimates and global signals were expanded with the inclusion of temporal derivatives and quadratic terms for each.^13^ Frames that exceeded a threshold of 0.5 mm FD or 1.5 standardized DVARS were annotated as motion outliers. Additional nuisance timeseries are calculated by means of principal components analysis of the signal found within a thin band (crown) of voxels around the edge of the brain, as proposed by Patriat et al.^14^ The BOLD time-series were resampled into standard space, generating a preprocessed BOLD run in MNI152NLin2009cAsym space. First, a reference volume and its skull-stripped version were generated using a custom methodology of fMRIPrep. All resamplings can be performed with a single interpolation step by composing all the pertinent transformations (i.e. head-motion transform matrices, susceptibility distortion correction when available, and co-registrations to anatomical and output spaces). Gridded (volumetric) resamplings were performed using antsApplyTransforms (ANTs), configured with Lanczos interpolation to minimize the smoothing effects of other kernels.^15^ Non-gridded (surface) resamplings were performed using mri_vol2surf (FreeSurfer). The presented description was generated automatically by fMRIPrep. which operates under the CC0 license and then modified by the authors of this work for clarity and accuracy.

# **Supplementary Text S2 Detailed statistical analysis**

This section provides a detailed overview of the statistical methods used for analyzing structural, ALFF, and functional connectivity data. Structural MRI metrics were analyzed using linear mixed-effects (LME) models to assess longitudinal changes and group differences. For each structural region, a separate LME model was constructed with subject ID as a random effect, and group, time, and their interaction as fixed effects. Age and sex were included as covariates in all models. Multiple testing across brain regions was addressed using the Benjamini-Hochberg false discovery rate (FDR) correction, with statistical significance set at p-FDR < 0.05.

Functional MRI analyses were conducted using the CONN toolbox (v22.a). We first performed whole-brain ALFF (Amplitude of Low Frequency Fluctuations) analysis to identify regions showing significant longitudinal or group-level effects. ALFF was computed by filtering the preprocessed BOLD time series to retain fluctuations within the 0.008–0.09 Hz range, and the amplitude of these fluctuations was estimated voxel-wise. Second-level group comparisons were conducted using mass-univariate GLM-based two-sided t-tests and F-tests, with age and sex included as covariates. Results were thresholded at voxel-wise p < 0.001 (uncorrected), and multiple comparisons were corrected at the cluster level using FDR at p-FDR < 0.05.

Based on the ALFF findings, ROI-to-ROI functional connectivity analysis was then performed on the subset of regions that showed significant ALFF differences. Fisher-transformed bivariate correlation coefficients were calculated between each ROI pair. Second-level group comparisons were performed using the same GLM framework, with age and sex included as covariates. Statistical maps were thresholded at connection-level p < 0.001 (uncorrected) and corrected using FDR at p-FDR < 0.05.

**Supplementary Text S3 Detailed information on handling and removing data**

To ensure data quality and reliability, all subjects were systematically excluded using predefined inclusion criteria. Following the extraction of brain regions from structural and functional data, a median-based imputation method was used to address missing values for specific regions. This method was selected as it can provide a reliable estimate of missing values without adding significant bias to the dataset. For the clinical, neuropsychological, and fluid markers, we used a selective approach: only subjects with available test results for the specific measures under investigation were included in the respective analyses and visualizations.

# **Supplementary Table 1 Longitudinal analysis of clinical and fluid biomarkers from baseline to follow-up 2 in post-COVID patients**

| **Variable** | **Estimate** | **Std. Error** | **Statistic** | **P-Value** | **Effect Size (Cohen’s *d*)** | **Change Direction** | **P Adjusted** |
| --- | --- | --- | --- | --- | --- | --- | --- |
| HADS anxiety | -0,9262852 | 0,38878147 | -2,3825342 | 0,02126426 | -2,3825342 | Decreasing | **0,03479606** |
| HADS depression | -0,3585203 | 0,36267511 | -0,9885439 | 0,32817098 | -0,9885439 | Decreasing | 0,40738467 |
| HADS total | -1,3222688 | 0,67841759 | -1,9490486 | 0,05749929 | -1,9490486 | Decreasing | 0,08999888 |
| PSQI | -0,6520779 | 0,51168255 | -1,2743798 | 0,20877996 | -1,2743798 | Decreasing | 0,26843138 |
| MoCA | 0,03542633 | 0,26504972 | 0,13365918 | 0,89398 | 0,13365918 | Increasing | 0,89398 |
| FSMC motor | -0,3795669 | 1,2734864 | -0,2980534 | 0,76705478 | -0,2980534 | Decreasing | 0,78897063 |
| FSMC cognitive | -1,2453615 | 1,34239036 | -0,9277193 | 0,35843551 | -0,9277193 | Decreasing | 0,43012261 |
| FSMC total | -1,8093168 | 2,61457671 | -0,6920114 | 0,49253775 | -0,6920114 | Decreasing | 0,57197932 |
| CRP | -30,082424 | 21,6118192 | -1,3919432 | 0,17385494 | -1,3919432 | Decreasing | 0,24072223 |
| Interleukin 10 | 1,50491439 | 0,08622437 | 17,4534686 | 6,3617E-19 | 17,4534686 | Increasing | **2,8628E-18** |
| Interleukin 2 | 0,73438125 | 0,05028954 | 14,6030626 | 3,1693E-27 | 14,6030626 | Increasing | **2,8524E-26** |
| Interleukin II-R. | 22,6106197 | 17,1927747 | 1,31512337 | 0,19540139 | 1,31512337 | Increasing | 0,26053518 |
| Interleukin 6 | 9,04835821 | 6,45217133 | 1,40237414 | 0,16643327 | 1,40237414 | Increasing | 0,24072223 |
| Interleukin 8 | 1,04999996 | 0,27110884 | 3,87298313 | 0,00309486 | 3,87298313 | Increasing | **0,00592628** |
| TNF alpha | 1,71105077 | 0,58229892 | 2,93844055 | 0,0091795 | 2,93844055 | Increasing | **0,01652311** |
| Ferritin | -30,741633 | 54,2906449 | -0,5662418 | 0,57462795 | -0,5662418 | Decreasing | 0,64645644 |
| NfL | -0,5791967 | 1,290551 | -0,448798 | 0,65537289 | -0,448798 | Decreasing | 0,70956542 |
| GFAP | -12,830207 | 29,9596216 | -0,42825 | 0,67014512 | -0,42825 | Decreasing | 0,70956542 |

# **Supplementary Table 2 Statistically Significant longitudinal changes in structural brain regions in post-COVID patients from baseline to follow-up 2**

| **Volume Measurement** | **Estimate** | **Std.Error** | **Statistic** | **p-Value** | **Effect Size (Cohen’s *d*)** | **Change Direction** | **p-Adjusted** |
| --- | --- | --- | --- | --- | --- | --- | --- |
| Left-Lateral-Ventricle | 270.0272249 | 59.44998574 | 4.542090658 | 1.80325e-05 | 4.542090658 | Increasing | 0.000486878 |
| Left-Cerebellum-White-Matter | -36.92369074 | 12.21205784 | -3.023543714 | 0.004355484 | -3.023543714 | Decreasing | 0.027380073 |
| Left-Cerebellum-Cortex | -186.1935536 | 46.56611712 | -3.998477115 | 0.000232975 | -3.998477115 | Decreasing | 0.003145167 |
| Left-Thalamus | -30.62255262 | 8.847218931 | -3.461263122 | 0.001155041 | -3.461263122 | Decreasing | 0.012437956 |
| Left-Caudate | -11.82271906 | 3.939060527 | -3.001405787 | 0.004563346 | -3.001405787 | Decreasing | 0.027380073 |
| Left-Putamen | -26.45845869 | 5.098910659 | -5.189041437 | 4.49968e-06 | -5.189041437 | Decreasing | 0.000161989 |
| Left-Pallidum | -9.236597911 | 3.373230192 | -2.738205632 | 0.008866652 | -2.738205632 | Decreasing | 0.045599927 |
| 3rd-Ventricle | 24.45270622 | 7.204355392 | 3.394156019 | 0.001476685 | 3.394156019 | Increasing | 0.013290163 |
| CSF | 11.90646155 | 3.571926806 | 3.333344213 | 0.001266829 | 3.333344213 | Increasing | 0.012437956 |
| Left-Accumbens-area | -6.142128282 | 1.562516522 | -3.930920536 | 0.000174703 | -3.930920536 | Decreasing | 0.00269542 |
| Left-VentralDC | -15.03040359 | 5.558418889 | -2.704078963 | 0.010162103 | -2.704078963 | Decreasing | 0.047717702 |
| Right-Lateral-Ventricle | 236.5041751 | 53.62458608 | 4.410368311 | 2.96232e-05 | 4.410368311 | Increasing | 0.000533217 |
| Right-Cerebellum-Cortex | -203.0699088 | 43.71984354 | -4.644799531 | 2.94037e-05 | -4.644799531 | Decreasing | 0.000533217 |
| Right-Thalamus | -28.72470081 | 9.152274773 | -3.138531297 | 0.003229668 | -3.138531297 | Decreasing | 0.023253611 |
| Right-Caudate | -11.65040114 | 3.314910942 | -3.514544234 | 0.000709911 | -3.514544234 | Decreasing | 0.008518933 |
| Right-Putamen | -26.02131983 | 4.493284566 | -5.791157771 | 1.67957e-07 | -5.791157771 | Decreasing | 1.81394e-05 |
| Right-Accumbens-area | -3.412650399 | 1.42850563 | -2.388965312 | 0.019123847 | -2.388965312 | Decreasing | 0.079437518 |
| Right-VentralDC | -16.51228122 | 5.990078501 | -2.756605146 | 0.008312794 | -2.756605146 | Decreasing | 0.045116062 |
| WM-hypointensities | 46.49371236 | 14.2118953 | 3.271464599 | 0.002018566 | 3.271464599 | Increasing | 0.016113141 |
| CC_Posterior | 4.567091306 | 1.729731303 | 2.640347259 | 0.009902535 | 2.640347259 | Increasing | 0.047717702 |
| CC_Mid_Anterior | -13.0065919 | 6.086633165 | -2.13691076 | 0.035515201 | -2.13691076 | Decreasing | 0.128369442 |
| ctx-lh-parstriangularis | -260447.893 | 88528.52845 | -2.941965687 | 0.004745747 | -2.941965687 | Decreasing | 0.799450571 |

# **Supplementary Table 3 Statistically Significant longitudinal clusters identified by ALFF analysis in post-COVID patients from baseline to follow-up 2**

| **Cluster (x,y,z)** | **Size** | **Size p-uncorrected** | **Size p-FDR** | **Brain Region** | **Voxel Count** | **Percentage Covering (%)** |
| --- | --- | --- | --- | --- | --- | --- |
| -8 +2 -2 | 4004 | 0.000000 | 0.000000 | Thalamus r | 234 | 18% |
|  |  |  |  | FOrb l | 211 | 13% |
|  |  |  |  | Pallidum l | 197 | 65% |
|  |  |  |  | Putamen l | 160 | 18% |
|  |  |  |  | Pallidum r | 155 | 58% |
|  |  |  |  | SubCalC | 147 | 13% |
|  |  |  |  | Thalamus l | 127 | 9% |
|  |  |  |  | FOrb r | 119 | 8% |
|  |  |  |  | Putamen r | 99 | 12% |
|  |  |  |  | Cereb6 r | 54 | 3% |
|  |  |  |  | toITG r | 47 | 6% |
|  |  |  |  | Amygdala l | 46 | 14% |
|  |  |  |  | pITG r | 44 | 5% |
|  |  |  |  | aPaHC l | 42 | 7% |
|  |  |  |  | Accumbens l | 40 | 37% |
|  |  |  |  | Accumbens r | 36 | 43% |
|  |  |  |  | Cereb1 r | 35 | 1% |
|  |  |  |  | TOFusC r | 31 | 4% |
|  |  |  |  | Hippocampus r | 29 | 4% |
|  |  |  |  | aPaHC r | 28 | 4% |
|  |  |  |  | Amygdala r | 25 | 7% |
|  |  |  |  | pTFusC r | 24 | 3% |
|  |  |  |  | Caudate l | 21 | 4% |
|  |  |  |  | Hippocampus l | 20 | 3% |
|  |  |  |  | Cereb2 r | 14 | 1% |
|  |  |  |  | pPaHC r | 10 | 3% |
|  |  |  |  | Cereb45 r | 8 | 1% |
|  |  |  |  | Cereb10 r | 6 | 4% |
|  |  |  |  | TP l | 5 | 0% |
|  |  |  |  | Caudate r | 4 | 1% |
|  |  |  |  | IC l | 2 | 0% |
|  |  |  |  | Cereb8 r | 2 | 0% |
|  |  |  |  | Brain-Stem | 1 | 0% |
|  |  |  |  | not-labeled | 1981 | 1% |
|  |  |  |  |  |  |  |
| +8 +40 +18 | 2560 | 0.000000 | 0.000000 | FP l | 496 | 7% |
|  |  |  |  | FP r | 382 | 5% |
|  |  |  |  | AC | 372 | 14% |
|  |  |  |  | PaCiG l | 249 | 19% |
|  |  |  |  | SFG r | 240 | 9% |
|  |  |  |  | SFG l | 197 | 7% |
|  |  |  |  | PaCiG r | 185 | 14% |
|  |  |  |  | SubCalC | 18 | 2% |
|  |  |  |  | not-labeled | 421 | 0% |
| +4 -26 -32 | 666 | 0.000000 | 0.000000 | Brain-Stem | 559 | 13% |
|  |  |  |  | Cereb3 r | 53 | 29% |
|  |  |  |  | Cereb3 l | 20 | 15% |
|  |  |  |  | Cereb45 l | 12 | 1% |
|  |  |  |  | Cereb45 r | 8 | 1% |
|  |  |  |  | pPaHC r | 3 | 1% |
|  |  |  |  | pPaHC l | 2 | 1% |
|  |  |  |  | aPaHC l | 1 | 0% |
|  |  |  |  | not-labeled | 8 | 0% |
| -52 -42 -28 | 267 | 0.000000 | 0.000000 | pITG l | 96 | 9% |
|  |  |  |  | Cereb1 l | 48 | 2% |
|  |  |  |  | toITG l | 32 | 5% |
|  |  |  |  | Cereb8 l | 19 | 1% |
| -4 -38 -34 | 100 | 0.000001 | 0.000000 | Brain-Stem | 99 | 2% |
|  |  |  |  | Cereb9 l | 1 | 0% |
| +26 -24 +58 | 90 | 0.000003 | 0.000000 | PreCG r | 82 | 2% |
|  |  |  |  | PostCG r | 6 | 0% |
|  |  |  |  | not-labeled | 2 | 0% |
| +52 +40 -12 | 82 | 0.000007 | 0.000000 | FP r | 62 | 1% |
|  |  |  |  | not-labeled | 20 | 0% |
| +38 -10 -44 | 65 | 0.000061 | 0.000001 | pTFusC r | 29 | 4% |
|  |  |  |  | aTFusC r | 14 | 5% |
|  |  |  |  | aITG r | 11 | 3% |
|  |  |  |  | pITG r | 2 | 0% |
|  |  |  |  | not-labeled | 9 | 0% |
| -32 -4 -38 | 56 | 0.000197 | 0.000005 | aTFusC l | 52 | 16% |
|  |  |  |  | pTFusC l | 2 | 0% |
|  |  |  |  | not-labeled | 2 | 0% |
| -26 -26 -24 | 52 | 0.000320 | 0.000009 | Hippocampus l | 26 | 3% |
|  |  |  |  | aPaHC l | 11 | 2% |
|  |  |  |  | pPaHC l | 6 | 2% |
|  |  |  |  | not-labeled | 9 | 0% |
| +36 +52 +12 | 47 | 0.000622 | 0.000018 | FP r | 47 | 1% |
| +58 -24 -20 | 41 | 0.001474 | 0.000047 | pMTG r | 13 | 1% |
|  |  |  |  | pITG r | 10 | 1% |
|  |  |  |  | not-labeled | 18 | 0% |
| +26 +14 -26 | 39 | 0.001887 | 0.000065 | FOrb r | 14 | 1% |
|  |  |  |  | TP r | 12 | 1% |
|  |  |  |  | not-labeled | 13 | 0% |
| -12 -28 -16 | 37 | 0.002442 | 0.000091 | Brain-Stem | 17 | 0% |
|  |  |  |  | pPaHC l | 3 | 1% |
|  |  |  |  | Cereb3 l | 3 | 2% |
|  |  |  |  | not-labeled | 14 | 0% |
| +18 -24 -10 | 35 | 0.003197 | 0.000128 | Brain-Stem | 18 | 0% |
|  |  |  |  | not-labeled | 17 | 0% |
| -52 -16 -26 | 33 | 0.004232 | 0.000181 | pMTG l | 13 | 1% |
|  |  |  |  | pITG l | 13 | 1% |
|  |  |  |  | not-labeled | 7 | 0% |
| -32 -56 +32 | 26 | 0.014135 | 0.000641 | sLOC l | 1 | 0% |
|  |  |  |  | not-labeled | 25 | 0% |
| +10 -42 +72 | 24 | 0.017605 | 0.000939 | PostCG r | 23 | 1% |
|  |  |  |  | Precuneous | 1 | 0% |
| -40 -24 +54 | 24 | 0.017605 | 0.000939 | PostCG l | 22 | 1% |
|  |  |  |  | PreCG l | 2 | 0% |
| -8 +32 +36 | 24 | 0.017605 | 0.000939 | PaCiG l | 21 | 2% |
|  |  |  |  | not-labeled | 3 | 0% |
| -50 +40 -12 | 23 | 0.020377 | 0.001141 | FP l | 21 | 0% |
|  |  |  |  | IFG tri l | 1 | 0% |
|  |  |  |  | not-labeled | 1 | 0% |
| -50 -14 +42 | 22 | 0.020861 | 0.001391 | PreCG l | 13 | 0% |
|  |  |  |  | PostCG l | 9 | 0% |
| -36 +42 +12 | 22 | 0.020861 | 0.001391 | FP l | 22 | 0% |
| +10 +18 +4 | 22 | 0.020861 | 0.001391 | Caudate r | 20 | 4% |
|  |  |  |  | not-labeled | 2 | 0% |
| +30 +48 -6 | 22 | 0.020861 | 0.001391 | FP r | 10 | 0% |
|  |  |  |  | not-labeled | 12 | 0% |
| -14 +26 -14 | 20 | 0.028959 | 0.002085 | MedFC | 1 | 0% |
|  |  |  |  | FOrb l | 1 | 0% |
| +6 -98 -8 | 19 | 0.033180 | 0.002566 | FP r | 20 | 0% |
| -40 -28 -20 | 19 | 0.033180 | 0.002566 | OP r | 15 | 1% |
|  |  |  |  | not-labeled | 4 | 0% |
| -54 +4 +24 | 18 | 0.039617 | 0.003169 | pTFusC l | 19 | 2% |
| +38 +48 +0 | 17 | 0.047541 | 0.003930 | PreCG l | 17 | 0% |
|  |  |  |  | not-labeled | 1 | 0% |

# **Supplementary Table 4 ROI Analysis of longitudinal functional connectivity changes in post-COVID patients**

| **Contrast** | **Source** | **Source Label** | **Mean Difference** | **Direction** |
| --- | --- | --- | --- | --- |
| **follow up 1  - baseline** | 11 | Default Atlas.Brain-Stem | -0,00143 | Negative |
|  | 4 | Default Atlas.Caudate l | 0,000163 | Positive |
|  | 3 | Default Atlas.Caudate r | 0,000426 | Positive |
|  | 12 | Default Atlas. Cerebelum Crus2 Right | 0,000995 | Positive |
|  | 10 | Default Atlas.Hippocampus l | 0,000264 | Positive |
|  | 9 | Default Atlas.Hippocampus r | 0,00053 | Positive |
|  | 8 | Default Atlas.Pallidum l | 0,000203 | Positive |
|  | 7 | Default Atlas.Pallidum r | 0,000329 | Positive |
|  | 6 | Default Atlas.Putamen l | -0,00025 | Negative |
|  | 5 | Default Atlas.Putamen r | -0,00017 | Negative |
|  | 2 | Default Atlas.Thalamus l | -0,00121 | Negative |
|  | 1 | Default Atlas.Thalamus r | -0,00136 | Negative |
| **follow up 2  - baseline** | 11 | Default Atlas.Brain-Stem | 0,000162 | Positive |
|  | 4 | Default Atlas.Caudate l | -0,00155 | Negative |
|  | 3 | Default Atlas.Caudate r | -0,00157 | Negative |
|  | 12 | Default Atlas. Cerebelum Crus2 Right | 0,000651 | Positive |
|  | 10 | Default Atlas.Hippocampus l | -0,00063 | Negative |
|  | 9 | Default Atlas.Hippocampus r | -0,00045 | Negative |
|  | 8 | Default Atlas.Pallidum l | -0,00071 | Negative |
|  | 7 | Default Atlas.Pallidum r | -0,00014 | Negative |
|  | 6 | Default Atlas.Putamen l | -0,00225 | Negative |
|  | 5 | Default Atlas.Putamen r | -0,00243 | Negative |
|  | 2 | Default Atlas.Thalamus l | -0,00146 | Negative |
|  | 1 | Default Atlas.Thalamus r | -0,00203 | Negative |
| **follow up 2 - follow up 1** | 11 | Default Atlas.Brain-Stem | 0,001594 | Positive |
|  | 4 | Default Atlas.Caudate l | -0,00171 | Negative |
|  | 3 | Default Atlas.Caudate r | -0,00199 | Negative |
|  | 12 | Default Atlas.Cerebelum Crus2 Right | -0,00034 | Negative |
|  | 10 | Default Atlas.Hippocampus l | -0,00089 | Negative |
|  | 9 | Default Atlas.Hippocampus r | -0,00099 | Negative |
|  | 8 | Default Atlas.Pallidum l | -0,00091 | Negative |
|  | 7 | Default Atlas.Pallidum r | -0,00047 | Negative |
|  | 6 | Default Atlas.Putamen l | -0,002 | Negative |
|  | 5 | Default Atlas.Putamen r | -0,00226 | Negative |
|  | 2 | Default Atlas.Thalamus l | -0,00025 | Negative |
|  | 1 | Default Atlas.Thalamus r | -0,00067 | Negative |

# **Supplementary Fig. 1 Stacked barplot showing symptom progression from baseline to follow-up 2 in post-COVID patients**

Count of patients (N=38) per symptom and category is shown. At each study visit a structured interview on respective symptoms and category of change was conducted. Grey = symptom was never present, dark magenta = symptom did not change, dark green = symptom improved, light magenta = symptom worsened, light green = symptom is no longer present, blue = symptom is new.

# **Supplementary Fig. 2 Correlation heatmap of structural and ALFF brain changes in post-COVID patients**. Correlation heatmap between structural and respective ALFF brain changes in post-COVID patients three years after infection. Statistical significance for correlation measures was determined using FDR correction (p < 0.05). Asterisks indicate statistically significant correlations (*p<0.05 and **p<0.01). Each cell in the heatmap represents a pairwise correlation between structural and ALFF measures using data from PCC patients with available three-year follow-up data

#

# **References**

1. Tustison NJ, Avants BB, Cook PA, et al. N4ITK: Improved N3 Bias Correction. *IEEE Transactions on Medical Imaging*. 2010;29(6):1310-1320. doi:10.1109/TMI.2010.2046908

2. Avants BB, Epstein CL, Grossman M, Gee JC. Symmetric diffeomorphic image registration with cross-correlation: Evaluating automated labeling of elderly and neurodegenerative brain. *Medical Image Analysis*. 2008/02/01/ 2008;12(1):26-41. doi:<https://doi.org/10.1016/j.media.2007.06.004>

3. Zhang Y, Brady M, Smith S. Segmentation of brain MR images through a hidden Markov random field model and the expectation-maximization algorithm. *IEEE Transactions on Medical Imaging*. 2001;20(1):45-57. doi:10.1109/42.906424

4. Dale AM, Fischl B, Sereno MI. Cortical Surface-Based Analysis: I. Segmentation and Surface Reconstruction. *NeuroImage*. 1999/02/01/ 1999;9(2):179-194. doi:<https://doi.org/10.1006/nimg.1998.0395>

5. Klein A, Ghosh SS, Bao FS, et al. Mindboggling morphometry of human brains. *PLoS Comput Biol*. Feb 2017;13(2):e1005350. doi:10.1371/journal.pcbi.1005350

6. Ciric R, Wolf DH, Power JD, et al. Benchmarking of participant-level confound regression strategies for the control of motion artifact in studies of functional connectivity. *Neuroimage*. Jul 1 2017;154:174-187. doi:10.1016/j.neuroimage.2017.03.020

7. Fonov VS, Evans AC, McKinstry RC, Almli CR, Collins DL. Unbiased nonlinear average age-appropriate brain templates from birth to adulthood. *NeuroImage*. 2009/07/01/ 2009;47:S102. doi:<https://doi.org/10.1016/S1053-8119(09)70884-5>

8. Jenkinson M, Bannister P, Brady M, Smith S. Improved optimization for the robust and accurate linear registration and motion correction of brain images. *Neuroimage*. Oct 2002;17(2):825-41. doi:10.1016/s1053-8119(02)91132-8

9. Cox RW, Hyde JS. Software tools for analysis and visualization of fMRI data. *NMR Biomed*. Jun-Aug 1997;10(4-5):171-8. doi:10.1002/(sici)1099-1492(199706/08)10:4/5<171::aid-nbm453>3.0.co;2-l

10. Greve DN, Fischl B. Accurate and robust brain image alignment using boundary-based registration. *Neuroimage*. Oct 15 2009;48(1):63-72. doi:10.1016/j.neuroimage.2009.06.060

11. Power JD, Mitra A, Laumann TO, Snyder AZ, Schlaggar BL, Petersen SE. Methods to detect, characterize, and remove motion artifact in resting state fMRI. *Neuroimage*. Jan 1 2014;84:320-41. doi:10.1016/j.neuroimage.2013.08.048

12. Behzadi Y, Restom K, Liau J, Liu TT. A component based noise correction method (CompCor) for BOLD and perfusion based fMRI. *Neuroimage*. Aug 1 2007;37(1):90-101. doi:10.1016/j.neuroimage.2007.04.042

13. Satterthwaite TD, Elliott MA, Gerraty RT, et al. An improved framework for confound regression and filtering for control of motion artifact in the preprocessing of resting-state functional connectivity data. *Neuroimage*. Jan 1 2013;64:240-56. doi:10.1016/j.neuroimage.2012.08.052

14. Patriat R, Reynolds RC, Birn RM. An improved model of motion-related signal changes in fMRI. *Neuroimage*. Jan 1 2017;144(Pt A):74-82. doi:10.1016/j.neuroimage.2016.08.051

15. Lanczos C. Evaluation of Noisy Data. *Journal of the Society for Industrial and Applied Mathematics*. 1964;Series B Numerical Analysis 1 (1): 76–85doi:<https://doi.org/10.1137/0701007>.
